# Supplementary material for: Temporal Changes of Fish Diversity and Driver Factors in a National Nature Reserve, China
Source: Animals (Basel). 2022 Jun 14;12(12):1544. doi: 10.3390/ani12121544 (PMC9219462; doi:10.3390/ani12121544)
Supplement: Supplementary file 1 [file animals-12-01544-s001.zip › Figure S1.pdf]

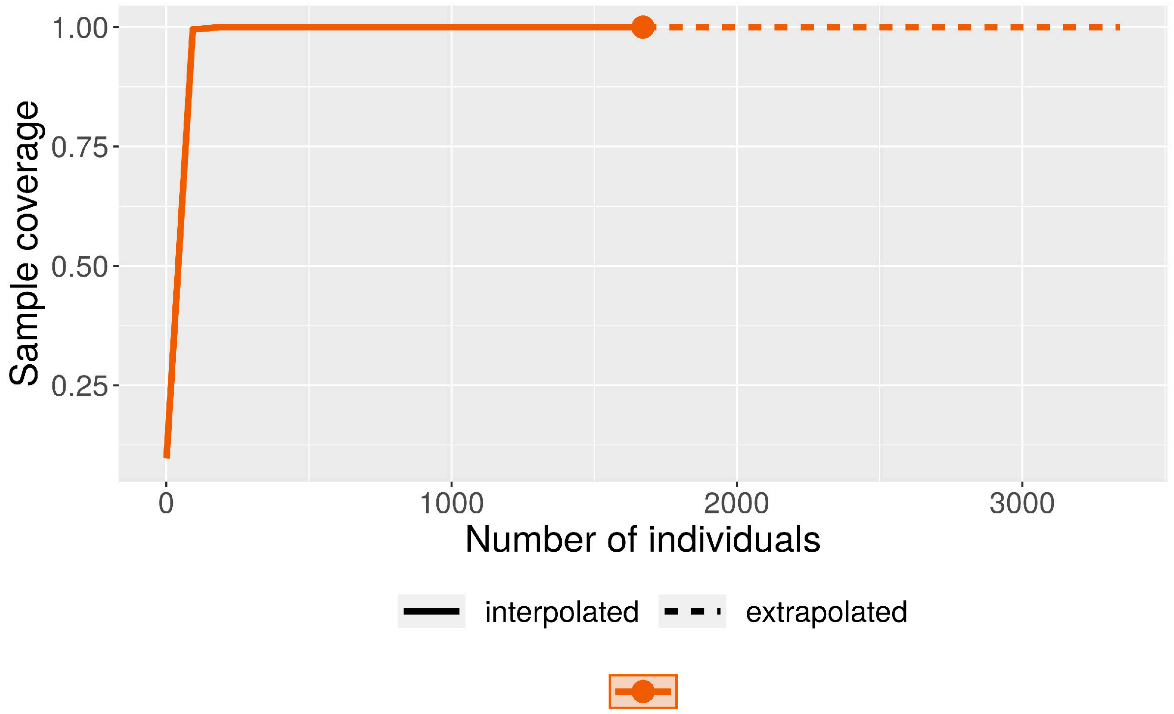

Figure S1. Species accumulation curves for fish at each area in the Lushan National Nature Reserve.Shaded areas represent the 95% confidence intervals.
