# Supplementary material for: Temporal Changes of Fish Diversity and Driver Factors in a National Nature Reserve, China
Source: Animals (Basel). 2022 Jun 14;12(12):1544. doi: 10.3390/ani12121544 (PMC9219462; doi:10.3390/ani12121544)
Supplement: Supplementary file 1 [file animals-12-01544-s001.zip › Table S1.pdf]

Table S1 The number of total, native and alien species in the Lushan National Nature Reserve in 2008 and 2021. The threatened species (i.e. CR, EN, VU or NT) are those according to threatened status based on Chinese Red-list results. Native species: Native to China; Alien species: Nonnative to China.

River	Number of order		Number of family		Number of genus		Number of species		Threatened species		Native species		Alien species	
	2008	2021	2008	2021	2008	2021	2008	2021	2008	2021	2008	2021	2008	2021
THY	2	2	2	2	5	4	5	4	0	0	5	4	0	0
GZ	3	3	4	4	9	5	9	5	0	0	9	5	0	0
XF	3	3	4	4	6	8	6	8	0	0	6	8	0	0
GYQ	2	3	4	5	6	9	6	9	0	0	6	9	0	0
GL	3	2	4	4	11	7	11	7	0	0	11	7	0	0
TY	3	2	4	2	10	2	10	2	0	0	10	2	0	0
JDX	3	3	4	4	5	6	5	6	0	0	5	6	0	0
LH	3	4	4	4	7	4	7	4	0	0	7	4	0	0
WJ	1	2	1	3	1	6	1	6	0	0	1	6	0	0
SMJ	2	4	3	7	5	8	5	8	0	0	5	8	0	0
WLQ	3	4	5	8	8	11	8	11	0	0	8	11	0	0
HH	2	4	4	5	6	7	6	7	0	0	6	7	0	0
LMG	—	2	—	2	—	2	—	2	—	0	—	2	—	0
OTM	4	—	5	—	5	—	5	—	0	—	5	—	0	—
