# Supplementary material for: Temporal Changes of Fish Diversity and Driver Factors in a National Nature Reserve, China
Source: Animals (Basel). 2022 Jun 14;12(12):1544. doi: 10.3390/ani12121544 (PMC9219462; doi:10.3390/ani12121544)
Supplement: Supplementary file 1 [file animals-12-01544-s001.zip › Table S2.pdf]

Table S2 Composition of fish catch in the Lushan National Nature Reserve in 2021

Species	*N*(%)	*W*(%)	*IRI*	Range of body length (cm)	Average body length (cm)	Range of weight (g)	Average weight (g)
Zacco platypus	72.16	56.09	11838.26	3.2-12.3	6.4	0.1-41.2	5.2
Rhinogobius giurinus	6.49	1.71	756.93	2.8-7.0	4.1	0.2-6.7	1.5
Acrossocheilus parallens	5.57	9.03	785.82	3.1-13.0	7.3	0.3-48.5	10.3
Opsariichthys bidens	4.54	9.27	530.84	4.7-16.2	9.0	1.9-61.3	12.8
Misgurnus anguillicaudatus	3.43	3.43	316.31	6.3-12.3	9.6	2.6-18.6	9.6
Liobagrus anguillicauda	1.72	1.52	174.56	4.0-9.5	6.4	0.7-14.0	5.5
Rhodeus ocellatus	1.66	0.41	31.88	2.1-5.6	3.8	0.3-4.0	1.5
Odontobutis sinensis	1.32	3.79	235.99	4.0-13.5	8.3	1.1-67.5	18.0
Monopterus albus	0.98	5.18	284.09	7.4-60.0	19.6	3.5-187.9	33.2
Carassius auratus	0.75	3.36	126.42	7.0-13.0	9.3	13.3-78.5	28.2
Pseudorasbora parva	0.69	0.62	40.17	4.8-9.3	6.5	1.6-14.6	5.6
Belligobio nummifer	0.69	1.87	39.40	4.6-13.2	10.0	1.5-33.3	17.0
Pseudobagrus ondon	0.63	1.02	38.21	4.7-12.2	8.2	2.0-22.0	10.1
Gnathopogon imberbis	0.34	0.51	6.60	6.0-9.1	7.6	5.0-15.8	9.3
Abbottina rivularis	0.11	0.23	2.68	5.0-12.0	8.5	2.0-23.4	12.7
Channa argus	0.11	1.96	15.93	10.5-25.5	18.0	17.0-196.2	106.6
